# Neuronal control of fixation and fixational eye movements

**DOI:** 10.1098/rstb.2016.0205

**Published:** 2017-02-27

**Authors:** Richard J. Krauzlis, Laurent Goffart, Ziad M. Hafed

**Affiliations:** 1 Laboratory of Sensorimotor Research, National Eye Institute, Bethesda, MD, USA; 2 CNRS, Aix-Marseille Université, UMR 7289 Marseille, France; 3 Werner Reichardt Centre for Integrative Neuroscience, Tuebingen, Germany

**Keywords:** fixation, eye movements, saccade, pursuit, microsaccade, superior colliculus

## Abstract

Ocular fixation is a dynamic process that is actively controlled by many of the same brain structures involved in the control of eye movements, including the superior colliculus, cerebellum and reticular formation. In this article, we review several aspects of this active control. First, the decision to move the eyes not only depends on target-related signals from the peripheral visual field, but also on signals from the currently fixated target at the fovea, and involves mechanisms that are shared between saccades and smooth pursuit. Second, eye position during fixation is actively controlled and depends on bilateral activity in the superior colliculi and medio-posterior cerebellum; disruption of activity in these circuits causes systematic deviations in eye position during both fixation and smooth pursuit eye movements. Third, the eyes are not completely still during fixation but make continuous miniature movements, including ocular drift and microsaccades, which are controlled by the same neuronal mechanisms that generate larger saccades. Finally, fixational eye movements have large effects on visual perception. Ocular drift transforms the visual input in ways that increase spatial acuity; microsaccades not only improve vision by relocating the fovea but also cause momentary changes in vision analogous to those caused by larger saccades.

This article is part of the themed issue ‘Movement suppression: brain mechanisms for stopping and stillness’.

## 1. Introduction

In humans and other primates, eye movements are made several times a second to place and hold images of objects of interest onto the central part of the retina, the fovea, where visual acuity is best. These eye movements fall into two main classes. Saccades are fast and brief movements, reaching peak speeds of hundreds of degrees per second and lasting only tens of milliseconds that quickly shift new visual images to the fovea. Smooth pursuit eye movements are slow and continuous, steadily rotating the eyes to track any motion of the visual target, minimizing the retinal slip and the resulting blur that might otherwise reduce spatial acuity. The neuronal mechanisms for generating saccades and pursuit have been studied in considerable detail, and involve circuits distributed across several cortical and subcortical brain regions [1]. Although important questions remain unanswered, these brain processes are understood well enough that saccades and pursuit are now routinely used as a benchmark for studying complex cognitive and perceptual issues.

Between these movements, the eyes are held relatively still. These periods of steady fixation have been studied much less frequently, and have often been treated as incidental pauses until the next eye movement is triggered. However, it is now clear that fixation itself is a dynamic process that appears to be actively controlled by many of the same brain structures involved in the generation of goal-directed eye movements. These periods are also crucial for analysing the visual image on the retina, which of course is often the reason for moving the eyes in the first place. In this article, we briefly review advances on several fronts. First, we point out that fixation does not occur in isolation, but is part of a larger oculomotor repertoire that includes saccades and smooth pursuit eye movements. Second, by reviewing the neuronal mechanisms for controlling fixation, we make the point that there is not a specific oculomotor subsystem dedicated to fixation, but instead fixation is simply an oculomotor context during which a target is kept on or near the fovea. Third, we describe how the eyes are not stationary during fixation but continue to move with a combination of several distinctive types of miniature movements. Finally, we discuss our emerging understanding of the impact that fixational eye movements have on visual perception.

## 2. Fixation and the decision to move the eyes

Starting an eye movement is often described as the outcome of a selection process that evaluates potential target locations in the visual field. This is only half the story. The transition from fixating to moving the eyes is not only determined by evidence in favour of the decision to move, but also by changes in signals that support the maintenance of gaze direction and inhibit the generation of unwanted eye movements. The interaction between these ‘go’ and ‘stay’ processes has been studied with behavioural tasks that are designed to manipulate these fixation-related signals.

**Figure 1.**
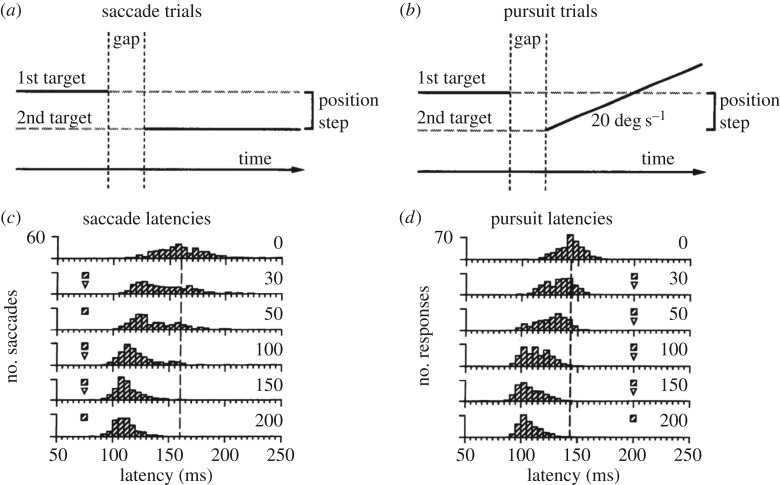
Gap effect on the latencies of saccade and pursuit eye movements. (*a*) Schematic of saccade trials. Human subjects initially fixated a target centred on the screen (1st target). After a temporal gap when no targets were visible, the second target appeared at an eccentric position. (*b*) On pursuit trials, the second target also appeared at an eccentric position and moved at a constant speed towards the centre of the screen. (*c*) Saccade latencies became shorter as the gap duration was increased from 0 to 200 ms. (*d*) Pursuit latencies decreased in a similar way as a function of gap duration. Figure adapted with permission from [2].

One example is the ‘gap paradigm’, which is a variant of the visually guided saccade task (figure 1*a*, left). In a standard visually guided saccade task, the peripheral target is turned on at the same time that the central fixated stimulus is turned off, and depending on the exact visual conditions, saccades are started at latencies of about 150–200 ms. In the ‘gap paradigm’, the central fixated stimulus is turned off earlier—about 200 ms before the peripheral target appears is usually the most effective [3,4]. This simple change has two effects on the latencies of saccades. First, the latencies of saccades overall are reduced by tens of milliseconds (figure 1*b*). Second, often but not always, there emerges a distinct set of saccades with much shorter latencies, referred to as ‘express saccades’. Conversely, the central fixated stimulus can be left on after the peripheral target has appeared, and this ‘overlap’ results in an increase in the latencies of saccades. These effects have been observed in many studies and illustrate that the decision to initiate a saccade depends on signals related to the fixated stimulus itself, including signals as simple as visual responses to such a stimulus (or its removal), as well as signals related to peripheral locations.

Another example is the ‘countermanding paradigm’ [5]. In this task, subjects are again asked to make standard visually guided saccades, but on a minority of trials, the fixation stimulus reappears shortly after being turned off, and this reappearance indicates to the subjects that they should cancel the saccade that they were in the process of starting. As might be expected, subjects fail to cancel the saccade if the foveal signal reappears too late, and by comparing the timing of this point of no return to the latencies of saccades, it is possible to estimate that the process of cancellation requires about 100–150 ms in humans [6–8] and somewhat less time in monkeys [9]. The success and failure at cancelling saccades in this task has been modelled as a race between an excitatory process driven by the peripheral target and a separate inhibitory process triggered by the reappearance of the foveal stimulus—the process that reaches its threshold first determines whether the saccade is cancelled or completed [6,8,10].

It is evident that saccades involve a break from fixation, but the situation is less obvious for smooth pursuit eye movements, which could be viewed as steady fixation of a moving stimulus—or conversely, fixation might be thought of as pursuit of a stationary stimulus. However, this equivalence has been ruled out by several studies showing differences between pursuing a moving target and fixating a stationary one. For example, if the position of a visual target is jiggled during fixation, the eyes do not follow. But if the target is jiggled during pursuit, the velocity of smooth pursuit eye movements is adjusted with remarkable sensitivity in an attempt to match the motion of the target [11,12]. This increase in sensitivity to target-related visual signals starts at pursuit onset and disappears as pursuit is stopped [13]. Thus, smooth pursuit also involves a break from fixation, which would otherwise prevent target-related motion signals from driving pursuit eye movements.

Given the above, the transition from fixation to pursuit has been studied by adapting the tasks used to study saccades, substituting a moving pursuit target for the stationary peripheral stimulus (figure 1*a*, right). In the ‘gap paradigm’, the reaction time for smooth pursuit shows the same dependence on gap duration as saccades, likewise being shortest when the fixation stimulus is turned off about 200 ms before the appearance of the moving target (figure 1*c*), in both humans [2,14] and monkeys [15]. However, unlike for saccades, the gap effect does not produce a separate population of very short latency pursuit responses. Also, with regard to triggering saccades, fixation and pursuit appear to have similar standing—the catch-up saccades that occur during smooth pursuit also show a ‘gap effect’ that has the same dependence on gap duration as those elicited during fixation [16]. Thus, changes in the foveal stimulus not only affect the timing of saccades to peripheral targets—these changes can also affect the decision to initiate smooth pursuit and the triggering of the small catch-up saccades that accompany smooth pursuit.

Similar conclusions follow from studies of smooth pursuit with the ‘countermanding paradigm’. In this case, it was found that the time required to cancel smooth pursuit is slightly shorter (by approx. 20 ms) than the time to cancel saccades. However, this difference is probably due to the fact that the generation of saccades, unlike smooth pursuit, includes a final ballistic-like interval that is not under inhibitory control [17,18]. When this interval is included in the calculations, the cancellation of saccades and smooth pursuit can be parsimoniously explained by a common inhibitory mechanism [18]. Further evidence is found in the effects of trial sequence during the task. The instruction to cancel a movement on one trial increases the latencies of saccades and smooth pursuit on the following trial [18], presumably because of residual activation of the inhibitory mechanism, and regardless of whether the previously cancelled movement was a saccadic or a pursuit eye movement.

These behavioural results illustrate the basic point that the decision to move the eyes depends on an interaction between signals from the foveal and peripheral parts of the visual field. Because this interaction is typically judged from the viewpoint of generating eye movements, the foveal, fixation-related signals appear to be inhibitory and act to prevent or delay the onset of the movement, reducing the rate at which the observer can scan their visual environment. However, the converse perspective is also valid—from the viewpoint of maintaining fixation, target-related signals in the periphery are inhibitory and act to shorten the period of fixation, reducing the time available to analyse the stimulus on their fovea. In some clinical conditions, but also in some healthy adults, subjects are prone to break fixation too easily, making it necessary to generate corrective saccades back to the previously foveated stimulus to get another look, resulting in a temporal pattern of eye positions called ‘square wave jerks’ [19]. Finally, situations have been documented in normal healthy subjects where a steady eccentric fixation is engaged, not towards the physical location of the target but towards a nearby location [20]. How does the interaction between fixation- and target-related signals normally take place, and what happens when it is disrupted? To address these questions, we next consider how our understanding of the underlying neuronal mechanisms has unfolded over time.

## 3. Neuronal mechanisms for controlling fixation

Neurons in several brain regions display the signatures of being involved in the control of fixation—elevated firing rates during maintained fixation and pauses during saccadic eye movements. The neurons closest to the motor output that are believed to be involved in fixation are the omnipause neurons. Located in the nucleus raphe interpositus of the paramedian pontine reticular formation, they exhibit a sustained firing rate during fixation and stop firing for saccades in all directions, regardless of their amplitude. Their tonic activity is presumed to prevent the generation of saccades by inhibiting the firing of saccade-related premotor burst neurons located in the mesencephalic and pontomedulllary reticular formations; the pause in their activity would allow the saccade-related burst to start and drive the motor neurons that innervate the extraocular muscles [21,22].

Because of their inhibitory influence on the saccade-related burst neurons driving both horizontal and vertical saccades, the omnipause neurons are commonly considered to be involved in fixation. However, this role should still be considered hypothetical, because there is no direct evidence that inactivation of omnipause neurons leads to problems with maintaining fixation [23]. Indeed, the possibility remains that their inhibitory influence only coordinates the horizontal and vertical components of saccades by synchronizing their onset.

Many omnipause neurons exhibit a small decrease in their activity during smooth pursuit eye movements [24,25]; this decrease may be part of the gating mechanism for smooth pursuit, but such a decrease could also reflect an aborted attempt to trigger a catch-up saccade that often occurs when a moving target is being tracked. The activity of omnipause neurons and their presumed role in gating the generation of saccades depends on inputs from several cortical and subcortical brain regions.

Among the structures that provide inputs to the omnipause neurons, arguably the best understood is the superior colliculus (SC), a multilayered sensorimotor structure located on the roof of the midbrain that is important for the control of saccades and other orienting behaviours. A defining feature of the SC is that it contains an eye-centred (i.e. retinotopic) map of the world, not only in the superficial layers that are involved in visual processing, but also in the intermediate and deeper layers that contribute to orienting movements. Across much of the SC map, neurons in the intermediate layers increase their firing rates when potential targets are present at particular eccentric locations in the visual field and exhibit bursts of activity for saccades with directions and amplitudes that bring that location to the fovea. These neurons are important for triggering saccades to peripheral targets. However, at the rostral end of the SC map, corresponding to the foveal region of the retinotopic map, neurons in the intermediate layers often show a complementary pattern of activity—elevated firing rates during fixation and pauses during most saccades, similar but not identical to the pattern exhibited by omnipause neurons [26,27]. These SC neurons also exhibit a neuronal correlate of the ‘gap effect’, decreasing their activity after the fixation stimulus is turned off with a time course that matches the behavioural effects on latencies for both saccades [28–31] and smooth pursuit [32]. These properties indicate a role in the control of steady fixation, and our understanding of how they contribute has changed over the past decade or so.

The original interpretation was that these SC neurons were ‘fixation cells' engaged in a push–pull interaction with saccade-related neurons elsewhere in the SC. According to this idea, mutual inhibition between neurons in the ‘fixation zone’ and the rest of the SC implemented a winner-take-all mechanism for determining whether fixation was maintained or a saccade was initiated. Experiments that manipulated the activity of so-called ‘fixation cells’ provided causal evidence in favour of this idea. Specifically, electrical microstimulation of the rostral SC increases the latencies of saccades to peripheral targets [33], as though a signal promoting fixation had been injected into the system. Conversely, reversible inactivation of the rostral SC by injecting pharmacologic agents produces difficulty in suppressing saccades to peripheral targets [33], as though the support for maintaining fixation had been degraded.

However, there are reasons to doubt the presence of a ‘fixation zone’ in the rostral SC. The anatomical connection from the SC to the omnipause neurons is not consistent with a distinct ‘fixation zone’—this projection arises from locations out to 10–15° in the SC map [34,35], which includes 80–90% of the saccades made during natural viewing [36]. Conversely, neurons across the entire SC are involved in the process of selecting targets for eye movements, and neurons in the rostral SC are especially important for the selection of targets near the fovea, even if this involves breaking visual fixation to move the eyes [37–39]. The receptive field and temporal properties of neurons in the rostral SC are in fact very similar to those elsewhere in the SC, except that these neurons represent the central visual field as expected from their location within the SC retinotopic map [40]. Even the initial descriptions of these neurons provided evidence that they were not a functionally distinct class of neurons, but were the extension of saccade-related neurons found elsewhere in the SC [26]. These findings indicate that the SC on the two sides of the brain do not contain antagonistic zones for ‘fixation’ versus ‘saccades’, but instead together form a single continuous map of the visual field in which the population of active neurons represents the locations of potential targets, including the one currently fixated [41].

If there is no ‘fixation zone’, then how is the break from fixation controlled? According to the more recently proposed ‘equilibrium hypothesis’, fixation corresponds to an equilibrium state in which target-related activity is balanced across the two SC, and saccades are triggered when this activity becomes sufficiently imbalanced [42,43]. To test this idea, activity in the rostral SC was locally suppressed by injecting a chemical agent, and the effects on visual fixation and saccades were measured [43]. If the rostral SC contained ‘fixation cells’ whose activity provided motor commands to maintain fixation, then suppression of this activity should lead to problems with maintaining fixation—there should be a major problem in stopping unwanted saccades. If, on the other hand, the SC contributed to fixation through a balance of potential target locations, then the ability to maintain fixation might still persist as long as an equilibrium was still possible.

**Figure 2.**
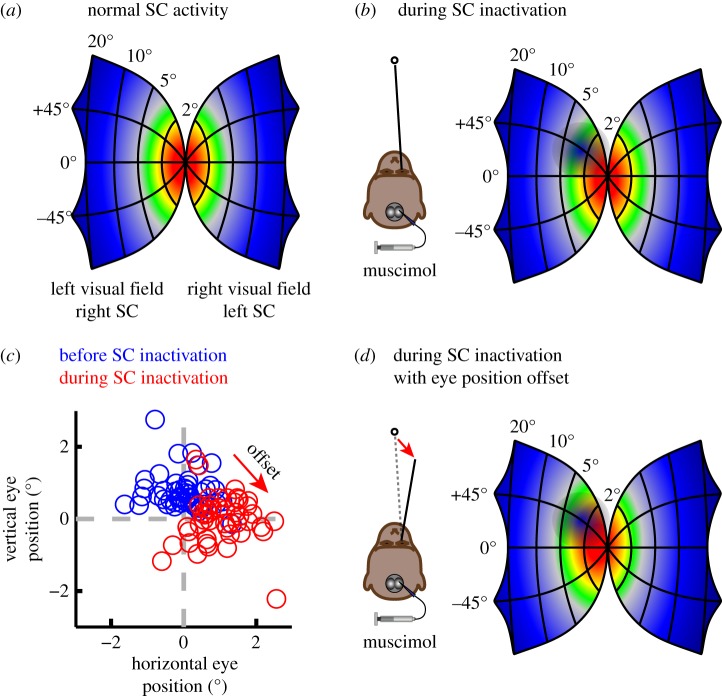
Eye position offsets caused by suppression of rostral SC activity. (*a*) Schematic of how neuronal activity is centred and balanced across the two SC during normal steady fixation. Higher activity is indicated by hotter colours. (*b*) During suppression of SC activity caused by injection of muscimol, a GABA_A_ agonist that inhibits neuronal activity, some of the neurons normally active during fixation are silent or less active, as indicated by the cool spot in the SC map. This change in population activity results in an imbalance in the activity across the two SC. (*c*) Sample data from a monkey subject (*Macaca mulatta*) showing the observed rightward and downward offsets in eye position caused by injecting muscimol into the right rostral SC during one experiment. (*d*) The stable offset caused by rostral SC inactivation can be explained by changes in how activity is distributed across the SC. As the eye position shifts down and to the right, the retinotopic distribution of activity also shifts, until a new equilibrium is reached with balanced activity across the two SC. Figure adapted with permission from [42,43].

The results provided clear evidence against the ‘fixation zone’ idea and in favour of the ‘equilibrium hypothesis’. Contrary to the ‘fixation zone’ idea, suppression of rostral SC activity did not impair the ability to maintain fixation. In fact, the occurrence of small saccades around the fixation stimulus decreased, possibly because of the reduction in SC activity evoked by the fixation target. Moreover, the results provided affirmative evidence in favour of the ‘equilibrium hypothesis’. According to this view, local suppression of the SC on one side should introduce a bias in the brain's internal estimate of the target location, because neurons normally used to code target location have been silenced on one side of the brain (figure 2). Consistent with this prediction, suppression of rostral SC activity caused a systematic and stable offset in eye position in the expected direction [43]. Similar effects also occur during smooth pursuit [42].

Can these observations be reconciled with the evidence that initially gave rise to the ‘fixation zone’ idea? Yes, by adopting the perspective that SC activity is not linked to a specific motor command to fixate or make a saccade, but instead is related to the location of the visual target. Thus, change in the activity of rostral SC neurons during the ‘gap paradigm’ is not a change in the motor command to fixate, but represents the presence or absence of a foveal target. Stimulation of the rostral SC delays saccades because it increases activity related to the foveal target, perhaps at the expense of activity representing the peripheral target, and makes it take longer to reach the tipping point for imbalance. Conversely, suppression of rostral SC activity results in irrepressible saccades to peripheral targets because signals representing the foveal target are weakened; notably, these irrepressible saccades do not occur in the absence of a competing peripheral stimulus [43]. Finally, a logical problem with the ‘fixation zone hypothesis’ is that, because the border between the ‘fixation’ and ‘saccade’ zones must lie at some location in the SC retinotopic map (typically assumed to be at approx. 2–3°), it predicts a lower bound on the size of saccades controlled by the SC. By contrast, because the ‘equilibrium hypothesis’ depends on the balance of activity across the SC, it provides a mechanism that scales across all saccade amplitudes, including microsaccades.

The exact direction of gaze during fixation is not determined by the symmetrical wiring between visual areas and the SC, but instead relies on bilateral activity in the medio-posterior cerebellum (MPC), a brain structure important for the adaptive control of gaze accuracy. In the case of orienting gaze towards a visual target (i.e. bringing a target's image within the foveal visual field), the vermal lobules VIc–VII and the caudal fastigial nuclei are important for fine-tuning the horizontal amplitudes of saccades so that gaze lands on the target, but recent work shows that their activity is also important for the control of fixation. Under normal conditions, bilateral activity from the MPC contributes to accurate control of gaze direction during fixation. When fastigial activity is altered, this not only alters the landing accuracy of saccades, but also introduces offsets in the direction of gaze during fixation [44–46]. During unilateral inactivation, the offsets during fixation result from the asymmetrical impairment of saccade amplitudes, and also from an altered encoding of foveal target position [47], similar to what is seen during suppression of activity in the SC [43]. It is important to note, however, that the offset is not an oculomotor disorder because the deviation of the eye in the orbit is contralateral when the head is free to move. In fact, the magnitude of the offset is identical regardless of whether the head is fixed or free, indicating an alteration in the equilibrium reached as gaze was directed towards the target [48].

The role of the SC and MPC in encoding the location of fixation has relevance for understanding the adaptation that often takes place in clinical cases of macular degeneration in the retina [49]. The degeneration creates a blind spot that often affects the central visual field, nullifying the normal advantage of fixating visual objects on the fovea. These patients often develop an eccentric ‘preferred retinal locus’ (PRL) for fixation that shifts the retinal image towards undamaged parts of the retina. The mechanisms that contribute to the development of the PRL are not understood, but it seems likely that circuits through the SC and MPC that encode the fixated target position play a central role.

Fixation-related signals have also been found in several other brain regions, although the details of how they contribute to the control of fixation are less well understood. Neurons in several areas of the cerebral cortex, such as parietal and frontal cortex [50–53], show elevated firing rates during fixation. These cortical areas—for example, the frontal eye fields—could contribute to the control of fixation through descending projections to the SC and precerebellar nuclei [54], acting through the mechanisms described above. Fixation offsets have also been reported during unilateral pharmacological perturbation of the frontal eye fields [55]. There are also frontal monosynaptic projections directly to the omnipause region in the brainstem [56], raising the possibility that the output from the cortex exercises independent control over fixation and the gating of eye movements at the final motor stages of processing.

## 4. Eye movements during maintained fixation

The term ‘fixation’ is misleading because gaze never settles into a completely motionless state during maintained fixation but instead exhibits tiny movements around the targeted location (also see [57]). A large fraction of these fixational eye movements are smooth but seemingly random changes in eye position. These smooth changes in eye position consist of two somewhat arbitrarily defined components—ocular tremor and ocular drift. Ocular tremor refers to higher frequency (40–100 Hz) oscillatory movements with very low amplitudes of about 6 arc-seconds (approx. 0.0017°). These movements are difficult to measure and consequently not studied that often [58]; this normal ocular tremor during fixation is also different from the larger and slower ocular tremor that occurs in patients with Parkinson's disease [59]. Ocular drift refers to lower frequency (less than 40 Hz) meandering movements that produce much larger shifts in eye position (many arc-minutes) and eye speeds up to about 1° s^−1^ [30]. These smooth changes in eye position in fixation have often been considered to be motor noise, possibly reflecting either stochastic firing patterns of motor units innervating the eye muscles [58] or fluctuations of extraocular muscle contractions. This explanation may be true for tremor, but ocular drift can also compensate for the miniature changes in head position that occur during normal fixation when the head is free to move, suggesting that the drift component can be actively controlled, at least under some circumstances [60].

Smooth ocular drift and tremor during fixation are punctuated at regular intervals by very small saccades called microsaccades. During maintained fixation, microsaccades occur several times a second on average [61], comparable with the rate at which larger saccades are made when scanning a visual scene. The amplitude of microsaccades reported in the literature has changed over time. The original descriptions reported microsaccades with amplitudes mostly smaller than 12 arc-minutes (0.2°), which are indeed miniature saccades [62–64]. However, partly due to the more widespread use of video-based eye trackers, subsequent studies often apply a more liberal criterion and include much larger saccades with amplitudes of up to about 1°, sometimes referring to them as ‘fixational saccades’.

Microsaccades have been treated as though they were a distinct class of eye movements, but the bulk of evidence indicates that they are mostly like other saccades, just much smaller. One key piece of evidence comes from the ‘main sequence’—the relationship describing how the peak speed of saccades increases with the amplitude of the movement, and which provides a signature of the underlying control mechanism [65]. The main sequence for microsaccades follows the same curve as that for larger saccades [66], indicating that both regular and miniature saccades are generated by the same motor control mechanism. Like larger saccades, microsaccades are also associated with accelerated post-movement drift speeds, and they tend to be anti-correlated in direction with post-movement drift direction, suggesting that microsaccades and drift interact synergistically [67]; these effects have some similarities with the post-saccadic enhancement observed for smooth pursuit [68], ocular following [69] and vergence eye movements [70].

One aspect that appears to be different between microsaccades and other saccades is the degree of voluntary control. Microsaccades are often described as involuntary movements, unlike larger saccades, which can be easily placed under voluntary control. However, several studies have demonstrated that microsaccades can be voluntarily controlled, at least to some degree. Their generation can be voluntarily suppressed, even in the absence of a visible target [71]. Similarly, when subjects perform a difficult visual task, their rate drops significantly [72,73], indicating that subjects can voluntarily suppress the generation of microsaccades as part of a strategy to improve task performance.

There is also evidence that the endpoints of microsaccades can be actively controlled. During fixation of a stationary stimulus, microsaccades tend to keep the eyes centred on the stimulus, counteracting the effects of ocular drift and reducing the mismatch between eye and target position [28,30]. During a virtual ‘needle threading’ task, microsaccades precisely relocate the line of sight alternately between the tip of the thread and the target, which were less than a degree apart [74]. Moreover, they can be directed so that the visual image falls not just onto the fovea, but more specifically onto the foveola, a very small subregion within the fovea [75]. These properties may depend on the presence of fine-grained visual features. Unlike larger saccades, it is not yet clear whether microsaccades can be voluntarily directed to specific locations in the absence of a visual target, but this may be due to the limited spatial precision of non-visual signals rather than a property of microsaccades themselves.

Consistent with these similarities, microsaccades and larger saccades are controlled by the same brain structures. Omnipause neurons in the brainstem pause during microsaccades just like they pause during larger saccades, except that the pauses are shorter [76–78]. Similarly, the population of saccade-related burst neurons in the brainstem that fire for larger saccades also burst for microsaccades [76,79]. The same regions of the cerebellum that are involved in fine-tuning the horizontal amplitudes of larger saccades are also involved in the adjustment of microsaccades [47,80].

**Figure 3.**
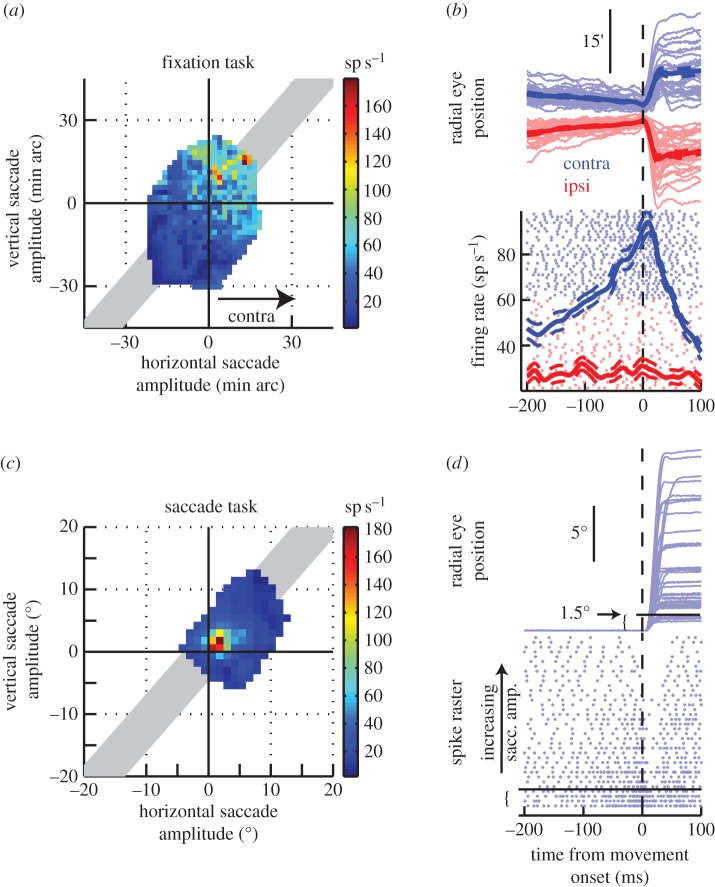
Sample neuron in the rostral SC showing activity related to microsaccades. (*a*) Movement-related activity of the neuron for all eye movements during fixation. Colour scale indicates the firing rate of the neuron during movements with different horizontal and vertical amplitudes; higher activity is indicated by hotter colours. The neuron exhibited preferential activation during small movements directed to the upper right quadrant. (*b*) Time course of microsaccade-related activity of the neuron. Radial eye position and firing rate are shown for contralateral movements along the preferred direction of the neuron (blue) as well as for ipsilateral movements along the same axis (red). The neuron exhibited build-up of activity for preferred movements. (*c*) Movement-related activity of the same neuron as in (*a*), but during larger visually guided saccades. The neuron exhibited increased activity for small saccades along the same preferred direction as in (*a*). (*d*) Time course of saccade-related activity. The neuron was active during saccades smaller than approximately 1.25°. For larger saccades, the neuron paused during the movements and the pause duration increased with increased saccade duration. Data recorded from a monkey subject (*Macaca mulatta*), and adapted with permission from [82].

The premotor control of microsaccades originates at least in part from the SC [40], and possibly the frontal eye fields [81]. Neurons in the rostral, foveal part of the SC map increase their activity before and during microsaccades with amplitudes of only a few minutes of arc (figure 3), and exhibit strong selectivity for the direction and amplitude of these movements [40,82]. Conversely, suppression of SC activity reduces the occurrence of microsaccades [40,43]. SC neurons with microsaccade-related activity show a distinctive build-up of activity that is very similar to that seen for neurons elsewhere in the SC map for larger saccades, consistent with the idea mentioned in §3 that the SC contains a single continuous retinotopic map that extends through the fovea (figure 3*b*). Many of these neurons also pause during larger saccades or saccades in a non-preferred direction (figure 3*d*), showing that these are the same neurons as those previously identified as so-called ‘fixation-cells’. Thus, consistent with the ‘equilibrium hypothesis’ described in §3, the control of microsaccades, saccades and fixation appears to depend on a population code in the SC representing the locations of possible targets.

The population code in the SC can also explain an interesting link between microsaccades and the cueing of covert attention. In both humans and monkeys, microsaccades can be precipitated by shifting visual attention to peripheral locations [73,83,84]; typically, the onset of a visual cue during fixation causes an increase in microsaccades in the direction of the cue location. These evoked microsaccades can be explained by changes in SC population activity—the activity at the peripheral site evoked by the cue creates an imbalance sufficient to trigger a saccade but not enough to draw a saccade to the peripheral location, hence the small saccade. Consistent with this interpretation, when SC activity at the cued location is suppressed, early microsaccades directed towards the cue are almost completely eliminated [85].

## 5. Perceptual consequences of ocular drift and microsaccades

Eye movements during fixation are not just a motor curiosity, but have substantial effects on visual perception and even on the control of other movements. The importance of these fixational eye movements on visual perception can be illustrated by stabilizing visual images on the retina as though the movements did not occur; this can be done experimentally by briefly flashing the stimulus and testing the retinal after-image, or by carefully measuring eye position and changing the visual display to match. During retinal stabilization, over several seconds, perception of the visual image fades and can even completely disappear [86,87]. By shifting where the visual image falls on the retina, fixational eye movements can contribute to avoiding such retinal fading [61]. However, image fading itself does not cause an increase in microsaccades [88]. Indeed, retinal fading is not normally a problem that needs to be solved—retinal images jitter all the time because the mechanisms for stabilizing gaze cannot eliminate all image motion, even when subjects are standing still [89].

During fixation, slow ocular drift is especially important for resolving fine spatial details. Using image stabilization, it is possible to selectively eliminate ocular drift as subjects briefly view orientated gratings. For stimuli with low spatial frequencies there is little effect, but for high spatial frequencies, eliminating ocular drift severely impairs the ability to judge the orientation of the visual stimulus [90]. Thus, under normal conditions, ocular drift improves the ability to visually discriminate high spatial frequencies. This effect involves interesting spatial and temporal transformations of the retinal input [91]. Drifting the image across the retina redistributes the power in the input image in favour of higher spatial frequencies; because natural images are dominated by low spatial frequencies [92], this redistribution in power has the effect of flattening or ‘whitening’ the input to the visual system. When viewing a stationary scene, ocular drift also shifts the temporal frequency of the visual input upwards into a range that can be detected with greater sensitivity by the retina.

Saccades during fixation have a different effect on the visual input. Fixational saccades, like larger saccades, shift the retinal image further and faster than ocular drift. Consequently, with regard to the visual input at the retina, fixational saccades add more power at low spatial frequencies than does ocular drift [93]. However, with regard to visual perception, the size of the saccade matters—visual sensitivity for low spatial frequencies is improved for fixational saccades larger than 0.5°, but there is no improvement in sensitivity for microsaccades [93]. Thus, unlike the case with ocular drift, the changes across spatial frequency caused by microsaccades may not substantially aid visual perception, although as mentioned above, microsaccades can improve spatial acuity by aligning fine-grained features with the foveola [74,75].

On the other hand, as part of the cost of moving the eyes, microsaccades temporarily interrupt visual processing, and invoke processes that help stitch together the continuous fabric of visual perception across each saccade. The interruption in vision during saccades is easily illustrated—if you look at the image of your face in a mirror, you cannot see your own eyes move as you make saccades. These brief lapses in visual processing that occur during each saccade, including microsaccades, are called ‘saccadic suppression’. In the laboratory, this effect typically appears as temporary impairments in the ability to detect or discriminate visual stimuli [94,95]. Saccadic suppression may be partly due to visual masking caused by moving the eye and rapidly changing the visual image on the retina [96]. However, this is not the complete explanation, because saccadic suppression affects the perception of visual stimuli presented not only during the movement, but also before the movement starts.

The neuronal basis of microsaccadic suppression has been examined in several brain regions, and the effects of saccades and microsaccades have been especially well documented for visual areas in the cerebral cortex. In general, visually responsive neurons in the cortex tend to show an enhancement of their firing rates in the wake of microsaccades [97], consistent with the sweeping of the stimulus across the neuron's receptive field that occurs during the microsaccade. These changes in the visual cortex are reproducible, but they do not provide a clear explanation for the suppressive effects of microsaccades on visual perception. By contrast, neurons in brain regions involved in the motor control of saccades show changes in firing rate that more tightly match the time course of perceptual changes during microsaccades. For example, the visual responses of visuomotor neurons in the SC and frontal eye fields are dramatically reduced if the visual stimulus is presented after microsaccades, but show enhancement if the visual stimulus is presented before microsaccades [98,99]. Thus, the gating of visual processing during fixational saccades may involve the same neuronal circuits that generate the movements themselves.

**Figure 4.**
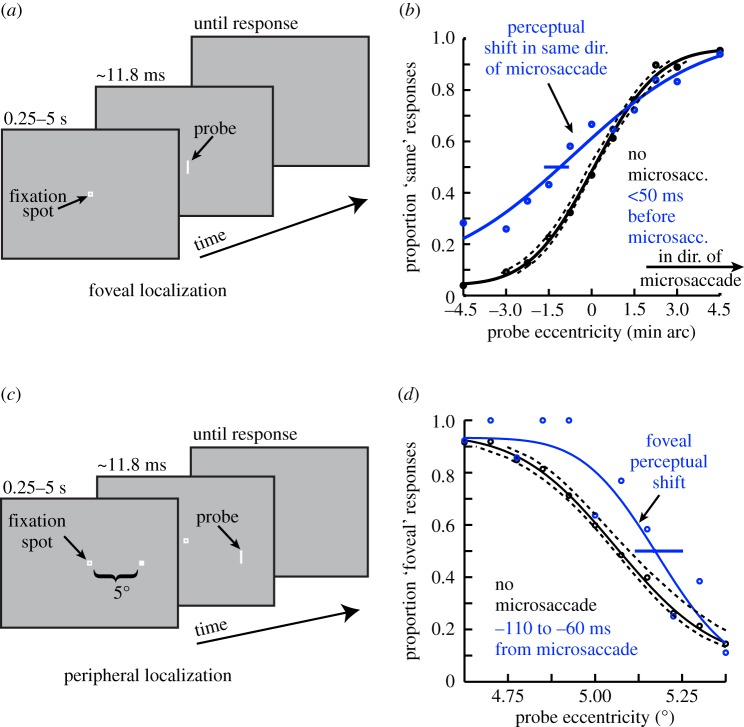
Mislocalization during microsaccades. (*a*) Foveal localization task used by [101]. Human subjects fixated, and a brief flash was presented at a variable horizontal displacement from the screen centre. (*b*) Perceptual localization performance in the task described in (*a*) when the briefly flashed probe was presented without microsaccades (black) or immediately before the onset of a microsaccade (blue). In baseline (black), the percept was veridical (the psychometric curve's point of subjective equality was at the point in which the probe was ambiguous in location). However, when the probe was presented before a microsaccade, the point of subjective equality was shifted such that subjects misperceived the stimulus as being displaced in the direction of the upcoming microsaccade. (*c*) Similar localization task but performed in the periphery. Subjects had to judge the location of a brief probe relative to a peripheral reference. (*d*) Mislocalization of the brief probe again occurred in the pre-microsaccadic interval, but it was now displaced foveally, opposite to the direction of the microsaccade. Figure adapted with permission from [101].

Microsaccades are also associated with distortions in the perception of object locations. It is well established that visual stimuli briefly flashed around the time of larger saccades are systematically mislocalized, mostly towards the endpoint of the saccade as though the visual field were compressed [100]. Recent results demonstrate that similar distortions of perceived locations also occur around the time of microsaccades [101]. When a foveal visual stimulus is flashed just before a microsaccade, it is mistakenly localized as displaced in the direction of the microsaccade (figure 4*a*,*b*). Conversely, when a peripheral visual stimulus is flashed, it is perceived as displaced towards the foveal endpoint of the microsaccade (figure 4*c*,*d*). The amplitude of the perceptual mislocalization with microsaccades is considerably smaller than the effects found with larger saccades, perhaps due to the much smaller size of the saccades involved, but the shared compression of the visual field suggests that similar mechanisms are involved in both.

Finally, because microsaccades alter visual processing and involve the same neuronal machinery as larger saccades, there is the possibility that microsaccades are a contributing factor to the behavioural effects found in classic visual and oculomotor tasks. For example, because there is a refractory period after saccades of all sizes [102], the occurrence of a microsaccade during fixation could impose a delay in starting a saccade to a peripheral target. This type of interaction could contribute to the saccade latency effects observed in standard oculomotor tasks, although there is conflicting evidence whether this is the case for the ‘gap effect’ [103,104]. The interactions between microsaccades and visual processing raise additional possibilities. As noted earlier, the sudden onset of a visual cue during a task is known to cause an increase in microsaccades [73,83,84], and the occurrence of microsaccades, in turn, modulates visual processing [98,99]. Visual cues are often used to study higher-level processes such as selective attention, but it is possible that some of the excitatory and inhibitory behavioural effects found with visual cues could be due to the interposition of microsaccades during the performance of the task [95,105].

## 6. Conclusion

Fixation is a dynamic process in several respects. First, fixation is actively controlled by neuronal mechanisms that include many of the same brain regions involved in the generation of voluntary eye movements. The SC, together with the MPC, determines both the location where the eyes should remain fixating, and whether a saccadic or pursuit eye movement should be initiated to look at another location. The maintenance of fixation is not controlled by a dedicated zone in the SC, but instead depends on the balance of target-related activity across the SC's retinotopic map as well as modulatory signals from the MPC. Second, the eyes are not stationary during fixation but continue to move with a combination of microsaccades, smooth ocular drifts and tremor. Microsaccades are the largest of these fixational eye movements and are controlled by the same neuronal mechanisms that generate larger saccades. Despite their small size, microsaccades can be voluntarily controlled to some degree, and involve the same neurons in the SC that are active during fixation. Third, eye movements during fixation have substantial effects on visual perception. By drifting the retinal image, smooth ocular drift transforms the visual input in ways that increase spatial acuity. Microsaccades can improve vision by relocating the fovea and foveola, but also cause momentary interruptions and visual distortions that are analogous to the disruptions also caused by larger saccades.

Thus, far from being a quiet interlude between eye movements, fixation is a lively period of continuous albeit microscopic motion, full of complex interactions that we are just beginning to understand. Some of the major outstanding problems to be solved include the following:

— Assuming that fixation is accomplished by an equilibrium of neuronal activity involving the SC and cerebellum, as indicated by current results, several key issues are unresolved. How is the equilibrium point reached so quickly, and how is the threshold for imbalanced activity implemented?
— The exact mechanism that reads out the population activity from the SC to maintain fixation or trigger a saccade is not understood. Does activity in the peripheral field of the SC have to be very strong or is it simply the locus of activity that matters? What computations take place at the level of the omnipause neurons and other neurons in the reticular formation as they integrate inputs from across the SC map?
— The mechanisms that generate microsaccades are much better understood than other fixational eye movements. What are the neuronal mechanisms that control ocular drift and tremor? Is ocular drift controlled by the same mechanisms in the SC and cerebellum that control fixation?
— Our understanding of fixational eye movements is advanced by—but also limited by—the particular methods used to measure eye movements [106,107]. Specifically, video eye trackers are now the most commonly used method, but are not well suited for studying fixational eye movements. Which advances in technology will make it possible for more investigators to routinely study fixational eye movements with the requisite precision?
— Foveal vision is a distinctive feature of primate vision but is difficult to study at the neuronal level, because fixational eye movements shift the very small foveal receptive fields. Will new techniques finally make it feasible to investigate the unique properties of foveal processing during visual tasks [108]?
— Conversely, how can our understanding of fixation be extended to other animal species that have an extended fovea (e.g. macula in cats) or those with little or no high-acuity retinal region? Are the processes for visually orienting towards tiny objects the same or different from those for orienting towards large objects?
— Most studies of fixational eye movements have been done with the head immobilized. How might the properties of fixational eye movements change when subjects are free to move their heads and move around more naturally as they interact with their surroundings?
